# Estimation of the Recent Expansion Rate of *Ruspolia nitidula* (Orthoptera) on a Regional and Landscape Scale

**DOI:** 10.3390/insects12070639

**Published:** 2021-07-14

**Authors:** Oto Kaláb, Petr Pyszko, Petr Kočárek

**Affiliations:** Department of Biology and Ecology, Faculty of Science, University of Ostrava, Chittussiho 10, CZ-710 00 Slezská Ostrava, Czech Republic; oto.kalab@osu.cz (O.K.); petr.pyszko@osu.cz (P.P.)

**Keywords:** spreading, climatic change, distribution, Orthoptera

## Abstract

**Simple Summary:**

Recent changes in insect distribution are consistent with the effects of climate and habitat change. The bushcricket *Ruspolia nitidula* has expanded in Western and Central Europe in recent decades. In the Czech Republic, *R. nitidula* was recorded in 2006 after ca. 50 years of absence. Using available occurrence data from professionals and citizens we estimated the *R. nitidula* expansion rate from 2006 to 2020 in the Czech Republic. For comparison, we monitored in detail expansion at the areal margin in the Odra River basin from 2016 to 2020. To estimate the expansion rates, we used three different methods of spatial analysis, including least-cost path analysis with habitat suitability. The estimated maximum expansion rate ranged from 13.8 to 16.2 km/year based on occurrence data at the country level and from 11.1 to 11.7 km/year based on the monitoring in the Odra River basin.

**Abstract:**

Recent changes in insect distribution are consistent with the expected interacting effects of climate and habitat change. The orthopteran *Ruspolia nitidula* has expanded its area of distribution in Western and Central Europe in recent decades. Because males emit a sound that is easily detected at a distance of up to 40 m, it is possible to detect spreading individuals and to therefore document routes and rates of spread. Using occurrence data at the landscape scale and three methods, including least-cost path analysis with habitat suitability, we estimated the *R. nitidula* expansion rate from 2006 to 2020 in the Czech Republic; this involved estimating distances between two origin occurrences in 2006 and two occurrences on the area margin in 2020. For comparison, we directly monitored expansion based on detection of singing males at the regional scale at the areal margin in the Odra River basin (2016–2020). The estimated maximum expansion rate ranged from 13.8 to 16.2 km/year based on occurrence data at the landscape scale and from 11.1 to 11.7 km/year based on the monitoring of males in the Odra River basin. To our knowledge, this is the first report of the direct monitoring of individual spreading males to detect changes in the distribution of an orthopteran.

## 1. Introduction

Recent changes in insect distribution are consistent with the expected interacting effects of climate and habitat change. There are many examples of high-latitude margins of insect distributions expanding polewards [1,2,3] and of contractions or local extinction at low-latitude or low-elevation range limits [4,5]. Global meta-analysis shows that insect distributions have, on average, shifted polewards and to higher elevations following geographic shifts in isotherms, and such poleward shifts are more rapid in the Northern than in the Southern Hemisphere (18.54 km per year northward shift in centroids) [6].

Distributional area changes and the current spreading of animals are usually detected based on the findings of newly established populations, because the spreading of individuals is usually below the limits of observability, especially in the case of insects and other terrestrial invertebrates. Most invertebrates are difficult to find because they are small, are difficult to identify, and are confined to specific ecological niches [7]. In the case of insects, changes in the areas of distribution are usually documented by random findings of individuals or by standardized monitoring schemes or citizen science projects [8]. Regardless, the history of spread remains mostly unknown in a given area.

Because of their sensitivity to both land-use and climate change, Orthoptera are generally useful for investigating the effects of recent environmental changes and their effects on range shifts [3,9,10]. In the current research, we selected the bushcricket *Ruspolia nitidula* as a model for studying the process of range expansion. *R. nitidula* has expanded its area of distribution in Western and Central Europe in recent decades (e.g., [11,12,13]). It is strong flier and therefore has the potential to spread over long distances [14,15]. There are currently many spreading species of Orthoptera (e.g., *Conocephalus discolor*, and *Phaneroptera* spp., and *Oecanthus pellucens*) (e.g., [3,16,17,18,19]), but *R. nitidula* has characteristics that facilitate the detailed mapping of its spread. *R. nitidula* males emit a characteristic sound, which is readily detectable at distances of tens of meters [15]. For this reason, it is possible to detect spreading individuals to map the routes in relation to the landscape topography but also to document the spread rates in a given environment.

*R. nitidula* is a good model for studying climate-induced range shifts due to good flying ability and free movement through the open non-forested landscape [11]. Many insect species are habitat specialists and need stepping stones, therefore the spreading could be influenced by the availability of optimal habitat or its fragments. There is limited knowledge about *R. nitidula* dispersal abilities, the authors only generally consider *R. nitidula* as mobile (e.g., [20,21]), capable of long-range dispersal several kilometers distant (e.g., [22,23]). No exact study has yet been devoted to flight capabilities, only Monnerat [11] supposed high mobility due to the recorded colonization of sites that were 10 to 15 km apart.

Expansion rates have traditionally been measured based on the occupancy of square grids and their geographical distances [24,25]. However, the geographical (i.e., Euclidean) distance represents the shortest possible path and does not account for the landscape matrix, which is one reason least-cost methods were introduced in the field of landscape ecology [26,27]. The purpose of least-cost analyses is to identify optimal routes regarding a raster matrix (i.e., a cost surface), which represents the ease/difficulty of dispersal for a species across particular parts of the landscape [26]. One option for creating a cost surface is to use habitat suitability models (but see, [28]). Least-cost analyses are widely used in ecology for identifying potential movement paths and corridors [29,30,31], and have application in the maintaining of habitat connectivity [32] and in the planning of greenways [33]. Although Mineur et al. [34] used least-cost path (LCP) analysis to measure distances to estimate range expansions of marine macrophytes, the LCP analysis was only used to avoid measuring the distance over the mainland, i.e., the coastline was recognized as a barrier that prevented movement of marine macrophytes.

The goal of our study was to estimate the rate of recent range expansion of the actively spreading bushcricket *R. nitidula*. The estimates are based on calculated distances from two sources of occurrence data that were obtained at different scales and with different sampling efforts. For one source (a landscape-scale source with uneven sampling), we used a national databases of documented records. For the second source (a regional-scale source with detailed, systematic sampling), we monitored the spread of *R. nitidula* for 5 years at the Moravian Gate. The Moravian Gate is a natural pass between the Sudetes and the Carpathians and is located on the areal margin of the *R. nitidula* distribution. Although the results of some studies indicated that the Moravian Gate currently plays no part in the south–north spread of some animals and plants [35,36,37], other studies have found that the Moravian Gate is an important ecological corridor [38,39].

We used three methods to estimate the distance of *R. nitidula* spread: geographic distance, length of the LCP, and length of the randomized-based LCP. We compare the estimates resulting from the analysis of the landscape-scale data that were obtained with an uneven sampling effort with the estimates resulting from the analysis of the regional-scale data that were obtained by systematic monitoring.

## 2. Materials and Methods

### 2.1. Model Species and Its Known Distribution

*Ruspolia nitidula* (Scopoli, 1786) (Orthoptera: Tettigoniidae) is a thermophilous bushcricket distributed in Southern and Western Europe, North Africa, and Western Asia [15,40]. In Europe, *R. nitidula* is widely distributed in the Mediterranean region. In Western Europe, its northern range reaches the Netherlands [13]. To date in Central Europe, the northernmost known localities of this species are in Germany, the Czech Republic, Slovakia, and Poland [12,15,41,42]. Mařan [43] reported the occurrence of this species in Pouzdřany and Věstonice in 1956. Since that time, no other occurrence has been recorded, even though suitable habitats are still common in this territory [44]. In 2006, the species began to spread from Austria and was recorded again at two sites in southern Moravia (Sedlec and Lanžhot). From that time, *R. nitidula* began to spread in the Czech Republic, and we now have information about occurrences across Moravia and a few sites in Bohemia. *Ruspolia nitidula* inhabits wet meadows, fens, riverbanks, and margins of ditches, where its eggs are laid, and larvae develop. Adults often migrate to dry places, e.g., dry meadows with high grassy vegetation, but also to the anthropogenic landscapes. Imagoes are found from the end of July to October [14,41,42]. Males stridulate mainly at dusk and at night; in doing so, the males create a loud buzzing sound that continues for minutes and that is interrupted by short high-pitched clicks [15]. The stridulation is so specific that it cannot be confused with any other animal sound.

### 2.2. Landscape-Scale Occurrence Data

**Figure 1:**
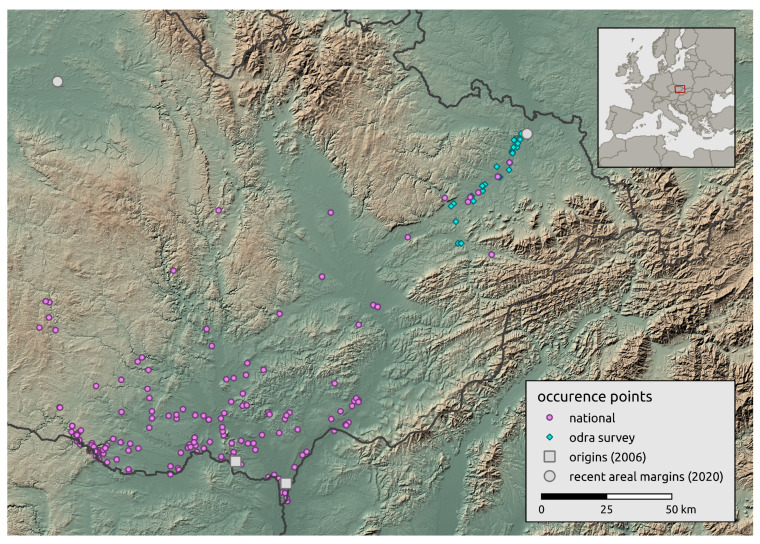
Overview of study area and *Ruspolia nitidula* occurrences. The occurrences include those in the national (landscape-scale) databases, and those obtained from the regional-scale survey in the Odra River basin.

We obtained landscape-scale occurrence data from the national database NCA CR [45], and occurrence data collected by professionals and citizens in the Czech Republic. Citizen observations were documented by photographs or recordings of stridulation. In total, we obtained 196 individual occurrence records for the landscape-scale occurrence of *R. nitidula*. Although these data were obtained by inconsistent sampling efforts, the initial recurrence sites (referred to as origin A and B) are well documented [44], and the two most distant recently known occurrences in 2020 are on the areal margins (one of them was added from survey data from Odra basin described in the next section) (see Figure 1).

### 2.3. Regional-Scale Field Survey at the Areal Margin

**Figure 2:**
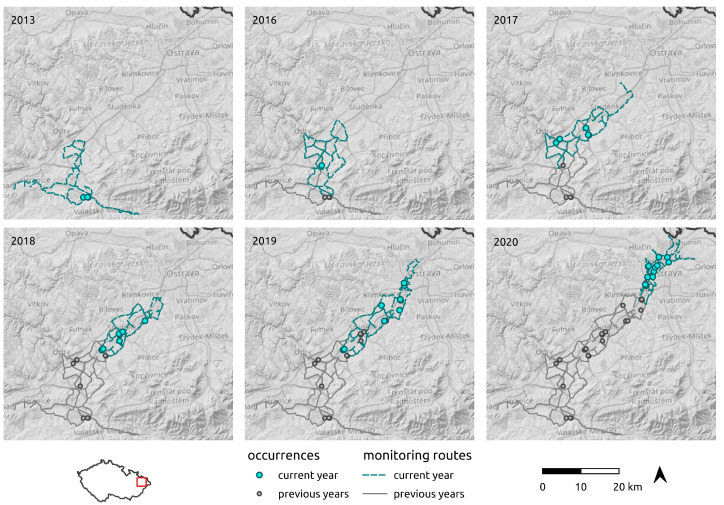
Driving routes used to monitor the spread of *R. nitidula* in the Odra River basin and *R. nitidula* detections in a 5-year period.

For the regional-scale survey, singing males were monitored acoustically from a car using a Pettersson D 200 ultrasound detector. The monitoring was conducted in August–September, on days with optimal weather conditions for *R. nitidula* singing (temperature at dusk > 18 °C, wind speed < 3.3 m/s^−1^ = 0–2 on the Beaufort scale). The first males were detected at the mouth of the Moravian Gate in 2013. Regular monitoring began in 2016 and was annually repeated until 2020 (the monitoring in each year is depicted in Figure 2). The monitoring began at dusk when the first males began to stridulate and continued to 22 h. The starting point was always in the northernmost area where *R. nitidula* males had been detected in the preceding season. After the first positive record of singing male, the observer started to monitor singing males by driving slowly through the landscape to the north with the windows opened on both sides at a speed of 20–50 km/h. If a male was detected, it was traced and located by triangulation method as precisely as possible. The position of the singing male was confirmed visually in most cases and the geographical position was recorded with a GPS Garmin eTrex Legend HCx with an accuracy 3 m. Males were audible at 20–40 m depending on the anthropogenic noise at the given location. During the monitoring, we recorded 42 individual occurrence records. We did not collect and preserve any voucher specimens, because each removed specimen could potentially affect subsequent spreading.

### 2.4. Dispersal Distances

We used three methods to calculate distances: geographical distance, LCP length, and passage LCP length. Geographical distances represent the shortest possible distance between occurrence points. LCP length, in contrast, indicates the single optimal route between occurrence points [46]. Passage LCP length includes a degree of randomness between LCP length and a random walk [46,47]. In estimating dispersal paths, both LCP methods take into account the cost surface based on a habitat suitability model (HSM).

A habitat suitability model was calculated with MaxEnt 3.4.3 [48] implemented in the R package *sdm* [49] via *dismo* [50]. We delimited the calibration area for model training on the Czech Republic boundary from the GADM dataset [51]. Based on known ecology and expert knowledge of the species, habitat suitability was assessed with the following seven landscape-related environmental factors: slope, terrain ruggedness, standard deviation of elevation, flat geomorphon type and compound topographic index (CTI) from Geomorpho90 [52], and Tree Cover Density and Grassland products (2018) from Copernicus Land Monitoring Service [53]. These variables were selected based on suitability for dispersal rather than on suitability for population persistence. Environmental raster data were cropped to national boundaries and resampled to ca. 15 arc second resolution. Preprocessed rasters were tested for multicollinearity with VIF using the *usdm* package [54] with a threshold of 0.7. We used all available occurrence records with localization uncertainty corresponding to the resolution of the raster, and we removed the oversampled occurrence clusters with 5-km distance spatial thinning with the *spThin* package [55]. This randomly removes the occurrences so that no occurrences are closer than 5 km to each other. We also removed three occurrences in central Bohemia (same locality in Čelákovice in 2011–2013) that were reported as known human introductions [56]. The population was established in the city garden in 2010 by 3 pairs transported from Hungary. The following year, several singing males were observed, and next year the temporary population spontaneously disappeared.

To optimize MaxEnt performance by choosing the best feature class and regularization parameter combination, we used the *ENMeval* package [57]. We ran a series of models with combinations of regularization parameters from 0.5 to 10 (by 0.5) with 8 feature class settings (“LQ”, “LQP”, “LQT”, “LQH”, “LQHT”, “LQTP”, “LQHP”, “LQHPT”; L = linear, Q = quadratic, H = hinge, P = product, T = threshold) with 5-fold cross-validation. We then selected the configuration with the lowest AICc (i.e., ΔAICc = 0) [58]. We ran 10 replicates of the model with 5-fold cross-validation. To evaluate model performance, we used the threshold-independent Boyce index [59,60] calculated with the *ecospat* package [61], and the threshold-dependent SEDI [62,63] calculated with threshold max(se + sp) with the script published in Wunderlich et al. [63]. MaxEnt AUC weighted ensemble output was used as a conductance surface for further LCP analyses.

In the analysis of the landscape-scale dataset, we calculated the distances from the two origin sites in 2006 [44] to all known occurrences, excluding data from the regional-scale field survey, except for one point delimiting the areal margin. We then used only the shorter distance of each site to one of the origin sites. Because we had detailed information about annual shifts in the Odra River basin (in the regional-scale dataset), in analyzing these data we calculated the stepwise distances for each year. We defined the origin site for each year as the point that had the lowest average distance to all sites discovered in the next year. In addition, in each step we removed the occurrences that were more distant than 3 km in the opposite direction (west) than the spread direction. We repeated this for every year.

Geographic distances (e.g., straight distances without regard to conductance surface) were calculated with the function pointDistance from the *raster* package [64]. To determine LCPs, we used the *shortestPath* function from the *gDistance* package [65]. For determining passage LCPs, we calculated randomized shortest paths [46] with the *passages* function in the *gDistance* package, which calculates average number of randomized shortest paths for each raster cell [65]. Passages were calculated separately for each pair of origin/other known occurrences, and the resulting raster was further used as cost surface to calculate a least-cost path. This approach enables calculation of single distance. The degree of randomness was defined by theta parameter θ = 0.0001. The transition matrix for analyses was calculated with mean function and connecting the cells in 16 directions [65].

### 2.5. Expansion Rate Analysis

In all cases, we filtered the resulting distances with a conditional maximum distance method through the years [25,34]. With this method, we obtained the cumulative maximum distance from the original points of recurrence, i.e., only the maximum distance from every year was used, and every maximum distance that was shorter than the maximum from previous years was replaced with the previous maximum [25]. Finally, we estimated the rate of expansion (km/year) as the slope of the simple linear regression of distance on year with the lm R function [25]. We also determined the distances from the origin sites in 2006 to the two recent areal margin occurrences in 2020 and divided the distances by the number of years to roughly estimate the average expansion of distribution per year. Because proper information about the spreading of the species through time at the landscape scale is lacking, we consider this as an estimate for comparison purposes. To determine whether the estimate of expansion rate on the landscape scale was affected by sampling effort, we randomly limited the data to 90%, 80%, 70%, 60%, 50%, 40%, 30%, 20%, and 10% of the occurrence reports (10,000 replications for each variant) and estimated the rate of expansion by each of the three methods. We fit the dose-response models from the package *drc* [66] to determine the relationship between expansion rate and sampling effort. Weibull’s four-parameter model was chosen as the best-fit for all methods based on the following criteria: the log likelihood value, Akaike’s information criterion (AIC), the estimated residual standard error, or the p-value from a lack-of-fit test. Based on the predictions of this model for 10-fold increase of sampling effort of original data, we determined whether the sampling effort was sufficient to provide a correct estimate of the expansion rate.

Analyses were conducted in R 3.6.3 [67], and spatial data were inspected, and maps were generated in QGIS 3.20 [68]. The code, QGIS project, and data to replicate the analysis are stored on GitHub (https://github.com/kalab-oto/ruspolia-expansion, accessed on 13 July 2021).

## 3. Results

### 3.1. Habitat Suitability Model

Two environmental variables were removed based on the results of the VIF multicollinearity test. Spatial thinning left 85 occurrence points for the model. The MaxEnt model with the lowest AICc (i.e., ΔAICc = 0) had a configuration with “LQP” feature classes and regularization multiplier 2. The model-dependent test means (±SD) were Boyce Index = 0.77 ± 0.10 and SEDI = 0.57 ± 0.09.

### 3.2. Landscape Scale

**Figure 3:**
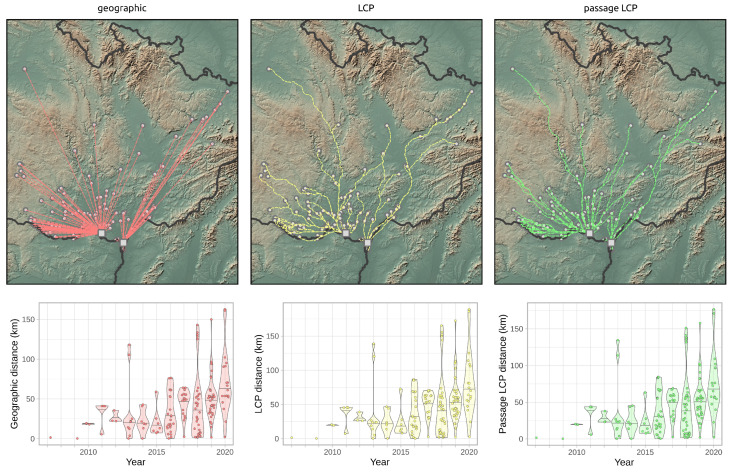
Paths of *R. nitidula* spread calculated with three methods for estimating distance (geographic, LCP, and passage LCP), and the distribution and median of their distances from 2007 to 2020. Squares in the maps represent two *R. nitidula* origin points.

**Figure 4:**
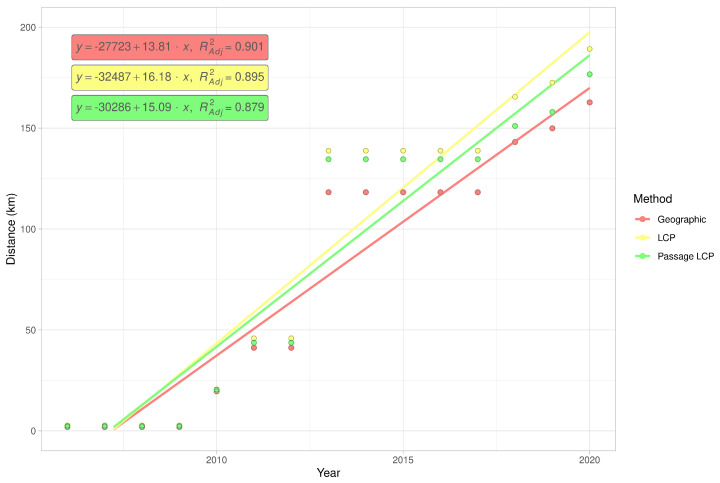
Simple linear regression of cumulative maximum distances of *R. nitidula* spread at the landscape scale, calculated with three methods for estimating spread distance: geographic, LCP, and passage LCP.

**Figure 5:**
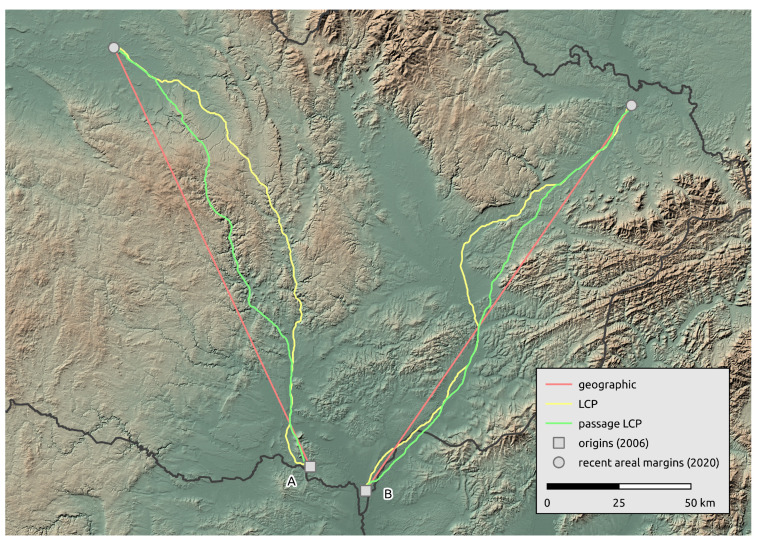
Comparison of *R. nitidula* spread paths from the two origin occurrences (2006) to the two most distant occurrences at the areal margin (2020) as calculated with three methods for estimating distance (geographic, LCP, and passage LCP).

**Figure 6:**
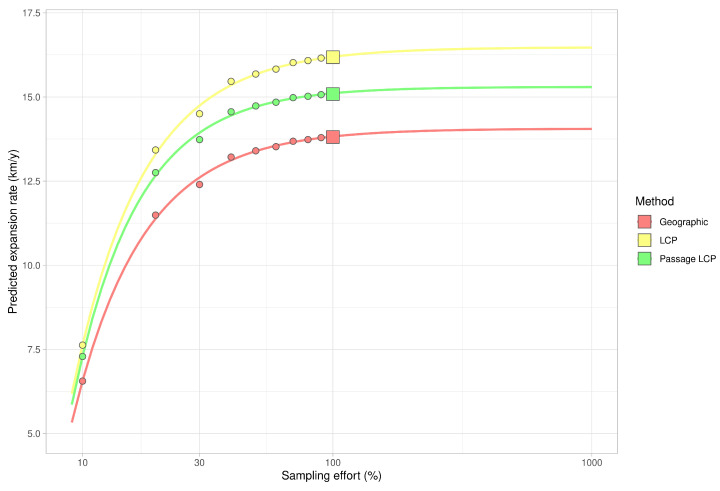
Estimates of expansion rate in decreasing and increasing sampling effort based on Weibull four-parametric dose-response model.

Calculated paths and distributions of their distances at the landscape-scale are shown in Figure 3. According to linear regression, the maximum estimated expansion rate at the landscape scale was 143.8 km/year (*p* < 0.001) with geographic distance, 16.2 km/year (*p* < 0.001) with LCP distance, and 15.1 km/year (*p* < 0.001) with passage LCP distance (Table 1, Figure 4). Explorative analysis of the two paths from the origin sites to the most distant recently reported sites showed average expansion rates of 11.5 km/year (origin A, 161.0 km) and 11.6 km/year (origin B, 162.7 km) with geographic distance, 13.5 km/year (origin A, 189.2 km) and 13.3 km/year (origin B, 186.1 km) with LCP distance, and 12.6 km/year (origin A, 176.7 km) and 12.2 km/year (origin B, 171.1 km) with passage LCP distance (Table 2, Figure 5). The estimated expansion rate reached 98.3% of the expansion rate predicted by the best fitting Weibull four-parameter model with geographic distance, 98.3% with LCP distance, and 98.6% with passage LCP distance; these results suggested that the landscape-scale sampling effort was sufficient (Figure 6).

### 3.3. Regional-Scale Survey in Odra Basin

**Figure 7:**
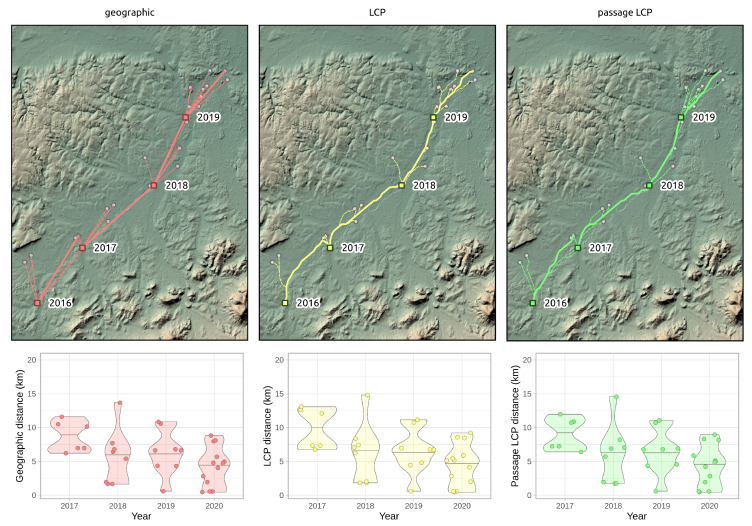
Annual paths of *R. nitidula* spread in the Odra River basin and distribution and median of distances as indicated by three methods for determining paths and distances (geographic, LCP, and passage LCP). Squares indicate origin points in a given year, and highlighted lines represent the path with the highest calculated distance.

**Figure 8:**
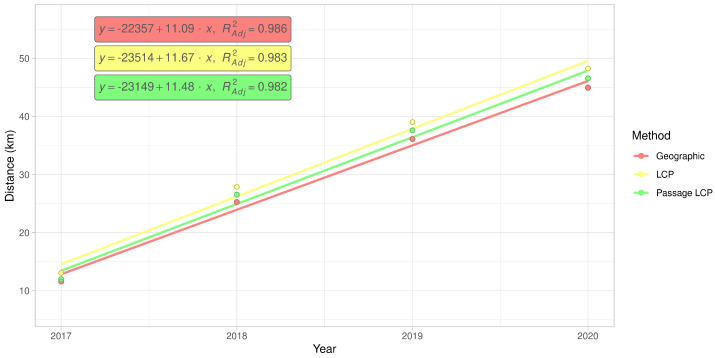
Simple linear regression of cumulative maximum distances of *R. nitidula* spread in the Odra River basin calculated with geographic, LCP, or passage LCP methods.

According to the slopes of the linear regressions of distance on year, the maximum expansion rate in the Odra River basin was 11.1 km/year (*p* < 0.01) with geographic distance, 11.7 km/year (*p* < 0.01) with LCP distance, and 11.5 km/year (*p* < 0.01) with passage LCP distance (Figure 7 and Figure 8, Table 3). Exploratory analysis of the specific annual distances is summarized in Table 4.

## 4. Discussion

After 50 years of absence, *R. nitidula* reappeared in the Czech Republic in 2006. In that year, the first individuals were detected in southern Moravia near the Czech–Austrian border. The species then began to continually spread to the northwest and northeast. Using three methods to calculate the spread distance, including two methods that accounted for habitat suitability (LCP and random LCP), we estimated the expansion rates and possible spreading routes.

According to the analyses of the maximum annual shift of all occurrence data at the landscape scale, the estimated maximum expansion rates was high (13.8–16.2 km/year). We are aware that this estimation of the overall expansion is partly based on spatially and temporally uneven occurrence data, and could therefore be biased. Moreover, the great disadvantage of the landscape-scale data is the effect of possibly insufficient sampling effort. We tried to assess this bias by estimating expansion rate on gradually randomly reduced data. Although reducing the dataset by 90% substantially reduced the estimated expansion rate (by 47–48% for all three distance methods), the prediction of the best-fit Weibull model for a 10-fold increase of sampling effort had virtually no effect on the expansion rate estimate (Figure 6); this suggested that the sampling effort was sufficient for these estimations. The estimates derived from the two longest paths (11.5–13.5 km/year) correspond better to linear model estimates and annual distances from our systematic field monitoring on the areal margin than to linear model estimates based on the landscape-scale data. During monitoring of dispersed males in the Odra River basin (2016–2020), we recorded the highest annual expansion between years 2017 and 2018. The distance estimated based on geographical distance was 13.7 km, and we consider this to be the lowest estimate of how far *R. nitidula* moves, i.e., we assume that the real distance is greater because the organisms are unlikely to move in a straight line. In contrast, the estimated distance based on LCP analysis was 14.8 km. The estimate using passage LCP analysis, which we consider more realistic than the estimate using LCP analysis, was only slightly lower than 14.6 km between these years. In other years, however, the annual distances moved were shorter (see Figure 7). Linear regression of maximum distances in our systematic survey provided estimates of expansion rates ranging from 11.1 to 11.7 km/year, depending on which method was used to calculate distance moved. Even with the mentioned unlikely LCP path between years 2017 and 2018, all three methods of distance calculation resulted in similar estimates. Moreover, the difference in calculated distances between the three methods did not change (Figure 8), unlike the distances at the landscape scale (Figure 4). We assume that this is caused by the openness of the landscape in the Odra River basin, which might be favorable for *R. nitidula* expansion.

As noted in the Section 1, *R. nitidula* has expanded its area of distribution in Western and Central Europe in recent decades and the supposed trigger is a warming trend (e.g., [11,12,13]). Simmons and Thomas [69] predicted range expansion to be characterized by two distinct phases. First, populations at the expanding margin should consist of large numbers of spreading individuals due to their selective advantage during the founding of new populations. Second, as additional populations in the landscape are established and the selective advantage of dispersal is reduced, the costs of expansion should select for lower dispersal rates, such that these populations should have characteristics of long-established rather than spreading population. Travis and Dytham [70] showed that these two phases of evolution should occur during expansion of species. The *R. nitidula* individuals monitored in the current study are apparently in the first phase of range expansion, i.e., the individuals are spreading and establishing temporary (or possibly permanent) populations that are the sources of next season’s migrants. We estimated possible spreading routes from two origin occurrences (2006) to two occurrences on the areal margin of the distribution (2020). In the northwest direction, the path goes from Morava and the Thaya conflux area through parts of the Dyje–Svratka Valley and Svitava Valley to the eastern part of Polabí; in the northeast direction, the path goes through the Lower Morava Valley, the Moravian-Silesian foothills, and the Moravian Gate to the Ostrava Basin. These modelled paths are unsurprising regarding the ecological requirements of the species. Although passive dispersal has been reported for orthopterans [71] and other insect species [72], and the most west-north occurrence in this study is isolated (Figure 1), we assume that the similar spread rate of the longest path on the contrary direction supports the plausibility that this occurrence record is the result of natural expansion. We also recognize that records west of the origin occurrences from 2006 may be the result of direct expansion from northern Austria (see [73,74]). Unfortunately, we could not examine this in detail, because precise data from Austria are not available. However, the first occurrence of Ruspolia in this area of the Czech Republic was recorded in 2011, and according to our regional survey estimates, the distance would be achievable by gradual expansion from origin occurrences reported in 2006. In any case, because we calculated our estimates only from a cumulative maximum distance from all data described in the Section 2.2, we assume that this will not have a significant effect on our estimates.

Climate-induced range shifts have been detected in many animals, and a significant portion of these shifts have been found in records collected over a long period of time [24]. There are various ways to calculate species expansions or shifts, but because detailed ecological knowledge for many species is missing, less complex methods based on species presence data are often used to assess distributional changes [25]. The majority of range shifts are detected by the simple comparison of recent and historical distributions. Hickling et al. [75] analyzed a 25-year dataset of 329 animal species in Britain and found that 275 of the species shifted northwards and 52 shifted southwards at their range margin; the latter study also reported that 22 species of Orthoptera had a northward shift at the range margin of 34 km during the 25-year period. Preuss et al. [25] evaluated the range expansion of the non-native bushcricket *Roeseliana roeselii* in Sweden using range margin statistics and the area-based method of grid occupancy. Both approaches resulted in estimates of spreading rates ranging from 1.5 to 3.0 km/year. However, Hochkirch and Damerau [76] reported that macropterous individuals of *R. roeselii* can disperse for distances up to 19.1 km/year. Simmons and Thomas [69] tested predictions for the evolution of dispersal during range expansion for four species of wing-dimorphic bushcrickets. The observed range expansion of macropterous *Conocephalus discolor* increased over time, from a rate of 1.22 km/year in the period 1976–1989 to 7.44 km/year in the period 1990–2002). Walker and Nickle [77] estimated the maximum spread rate of the mole crickets *Scapteriscus vicinus* and *S. acletus* at 20 km/year. Our study included the analysis of the maximum annual shift of occurrence data from long-term, landscape-scale observation and a 5-year annual systematic monitoring of dispersed males in the Odra River basin. Our use of direct monitoring of spreading males to detect annual range shifts is unique among Orthoptera, because we monitored spreading individuals before they established populations that are usually used for range shift estimation. Spreading individuals can provide clearer insight on used routes than reconstructions based on the established populations, those existences depend on the quality and position of a suitable breeding habitat.

One of the main pitfalls of using LCP analysis is a deterministic path calculation that leads to a single optimal path [46]. The known limitations and shortcomings of the use of LCP analysis in connectivity modelling include the assumption that moving individuals have knowledge of the entire landscape and the destination locality and will therefore follow only the one optimal path; on the other hand, LCP analysis introduced path stochasticity in the estimates of connectivity [27,78]. We suggest that these assumptions may not be so limiting in the context of calculating the rate of expansion, because we did not aim to describe the ability of an individual to travel a certain path (as in connectivity modelling), but instead aimed to assess the overall range expansion rate of the species. However, one optimal path generated with LCP analysis is problematic also in this case. Because LCP analysis relies on a cost surface, different input surfaces result in different routes. Researchers previously reported that distances of LCP analysis based on habitat suitability depend on the chosen HSM [79]. We therefore used the randomized shortest path (i.e., passage LCP analysis) as an alternative to geographic distance and LCP analysis. On the two longest paths (Figure 5), the lines representing geographic distances ignore unsuitable regions for *R. nitidula* (e.g., mountainous areas) and therefore estimated the shortest distance travelled per year. Paths estimated by LCP analysis, in contrast, strictly followed the most suitable places (i.e., pixels on the provided cost surface), and could overestimate the expansion rate. In the case of our regional-scale monitoring, the longest path 2016–2017 generated with the LCP analysis (Figure 7), leads to different point than other two methods, resulting in higher calculated distances. Additionally, in our preliminary analysis (unpublished data), we include altitude in the HSM, (which showed majority relative variable importance among other variables) and further least-cost path between the most distant northwestern occurrence and origin points followed the lowest possible altitudes and greatly circumvents the highlands ignoring its valleys, which results in measured distance of ca. 250 km.

Based on our results, we recommend caution in the use of LCP analysis to estimate an insect’s rate of expansion. On the other hand, use of geographic distance will always underestimate the rate to some extent. The difference in the estimated distances depend on the overall distance of expansion (as indicated by the diverging trend lines in Figure 4) and presumably on the impermeability of the landscape for the specific species. We suggest that adding some degree of randomization to the analysis, as is done with passage LCP, can provide more ecologically reasonable results than those obtained with the use of geographic distance or LCP analysis. Additional research is needed to determine which level of randomization is appropriate and the degree to which the suitability of the methods depend on the specific organism and landscape.

Recent climate-induced range shifts have been detected in many insect taxa in Central Europe, and *R. nitidula* seems to be a good model for the detailed evaluation of spreading paths and expansion rates due to good flying ability and free movement through the non-forested landscape. LCP analysis has indicated potential corridors for the spread of native or alien thermophilic species in association with climate warming. The expansion rate of *R. nitidula* documented in the current study indicates a natural and rapid shift northward of the areal margin across the entire width of the Czech Republic. Similar expansion rates can be expected for other insects that are strong fliers and that can freely move through open non-forested landscapes.

## Figures and Tables

**Table 1:** Results of simple linear models of the *R. nitidula* expansion spread rate at the landscape scale based on three methods for calculating spread distance.

Method	km/y	Adjusted R^2^	F	CI 2.5	CI 97.5	*p*-Value
Geographic	13.81	0.90	119.11	11.05	16.57	<0.001
LCP	16.18	0.90	112.31	12.86	19.51	<0.001
Passage LCP	15.09	0.88	95.07	11.71	18.46	<0.001

**Table 2:** Estimates of *R. nitidula* expansion rate based on distances between the two origins (2006) and the most distant recent occurrences (2020) calculated with three methods.

Origin	Method	km	km/y
	Geographic	161.0	11.5
A	LCP	189.2	13.5
	Passage LCP	176.7	12.6
	Geographic	162.7	11.6
B	LCP	186.1	13.3
	Passage LCP	171.1	12.2

**Table 3:** Results of simple linear models of rate of *R. nitidula* expansion in the Odra River basin based on three methods of distance calculation.

Method	km/y	Adjusted R^2^	F	CI 2.5	CI 97.5	*p*-Value
Geographic	11.09	0.99	209.64	7.80	14.39	0.005
LCP	11.67	0.98	169.61	7.81	15.52	0.006
Passage LCP	11.48	0.98	165.83	7.65	15.32	0.006

**Table 4:** Calculated *R. nitidula* spread distances (km) and their means ( ± SD) in the Odra River basin based on three methods of distance calculation.

Method	2016–2017	2017–2018	2018–2019	2019–2020	Mean ± SD
Geographic	11.60	13.67	10.85	8.83	11.24 ± 2.00
LCP	13.08	14.82	11.14	9.21	12.06 ± 2.42
Passage LCP	11.99	14.57	11.06	8.96	11.64 ± 2.32

## Data Availability

The data presented in this study are partially publicly available [45,51,52,53]. The rest of the used data, scripts for the analysis and visualizations are available on GitHub (https://github.com/kalab-oto/ruspolia-expansion, accessed on 13 July 2021). Part of the occurrence data is there provided in anonymized form (only coordinates and years) due to insufficient permissions.
